# Validity and Reliability of the European Organization Research and Treatment of Cancer Quality of Life Questionnaire-Oesophagogastric 25 in Indian Patients With Corrosive-Induced Benign Refractory Esophageal Strictures

**DOI:** 10.7759/cureus.37190

**Published:** 2023-04-06

**Authors:** Toshali Pandey, Ujjwal Sonika, Ashok Dalal, Ajay Kumar, Raghav Gera, Harshita Choudhary, Sanjeev Sachdeva, Siddharth Srivastava, Barjesh C Sharma

**Affiliations:** 1 Gastroenterology, Maulana Azad Medical College, New Delhi, IND; 2 Gastroenterology, Govind Ballabh Pant Institute of Postgraduate Medical Education and Research, New Delhi, IND

**Keywords:** burns chemical/psychology, caustics/adverse effects, esophageal stenosis/chemically induced, psychometrics, quality of life

## Abstract

Background

The European organization Research and Treatment of Cancer Quality of Life Questionnaire-Oesophagogastric 25 (EORTC QLQ-OG 25) is designed for patients with esophagogastric cancer. Its performance has never been tested with benign disorders. A health-related quality-of-life questionnaire does not exist for patients with benign corrosive-induced esophageal strictures. Hence, we evaluated the EORTC QLQ-OG 25 in Indian patients with corrosive strictures.

Methods

The English or Hindi version of QLQ-OG 25 was administered to 31 adult patients undergoing outpatient esophageal dilation at GB Pant hospital, New Delhi. These patients had refractory or recurrent esophageal strictures due to corrosive ingestion and had not undergone reconstructive surgery. Score distribution was analyzed, and item performance was determined based on floor and ceiling effects. Convergent validity, discriminant validity, and internal consistency were checked.

Results

The average time to finish the questionnaire was 6.70 minutes. Most scales fulfilled convergent validity (corrected item-total correlation >0.4), barring the Odynophagia scale and one item of the Dysphagia scale. Most scales exhibited divergent validity except for odynophagia and one item of dysphagia. Cronbach's alpha was >0.70 for all scales except odynophagia. Responses to questions evaluating taste, cough, swallowing saliva, and talking were highly skewed and had prominent floor effects. Overall, the questionnaire demonstrated good internal consistency, convergent validity, and divergent validity in benign corrosive-induced refractory esophageal strictures patients.

Conclusion

The EORTC QLQ-OG 25 can be satisfactorily used in patients with benign esophageal strictures to assess health-related quality of life.

## Introduction

The European organization Research and Treatment of Cancer Quality of Life Questionnaire-Oesophagogastric 25 (EORTC QLQ-OG 25) is a health-related quality-of-life (HRQL) module developed by the European organization Research and Treatment of Cancer to assess the quality of life in patients with upper gastrointestinal cancer [1,2]. The questionnaire utilizes patient-reported outcomes based on symptoms relevant to patients undergoing palliative or curative treatments and follow-up. Two studies from France have used this questionnaire for quality of life (QoL) measurement in patients with corrosive-induced strictures [3,4]. It has never been utilized in Indian patients with corrosive-induced strictures.

Corrosive ingestion poses a significant healthcare burden in developing countries [5-7]. A typical long-term sequela of corrosive ingestion is a refractory esophagus stricture. A stricture developing after a caustic ingestion is more likely to become complex and refractory than other etiologies of benign strictures [5,8]. The management of corrosive ingestion, especially in the long term, is unclear due to limited large studies. This leads to uncertainty and variations in management strategies worldwide [8]. Patients with benign refractory strictures are often treated with several years of periodic endoscopic dilation [9,10]. Some studies have revealed that long-term endoscopic management of corrosive-induced refractory strictures can have good outcomes from a clinical standpoint [11,12]. The definitive management for refractory or recurrent esophageal strictures is reconstructive surgery. However, no cut-off value has been established for the number or duration of such dilations before the modality is considered unsuccessful or reconstructive surgery is recommended [13-15]. Reconstructive surgery is technically demanding, leading to restricted access in developing countries [5,7,13]. The quality of life of individuals with corrosive-induced refractory esophageal strictures is subject to several factors. The event of corrosive ingestion itself is highly stressful and often performed as a suicidal act in adults [8]. During the chronic phase, individuals with refractory strictures suffer from dysphagia, aspiration, malnutrition, social isolation, multiple hospital visits, and a poor quality of life [3,16,17]. Thus, there is a need to gather patient-reported outcomes to help clinicians decide whether repeated endoscopic dilation of corrosive-induced esophageal strictures is appropriate and beneficial to patients.

No recommended tool for measuring the quality of life following corrosive ingestion exists. The 25-item EORTC QLQ-OG 25 questionnaire has six symptom scales (dysphagia, eating, reflux, odynophagia, pain and discomfort, and anxiety) and ten single items that address many of the problems faced by this patient population. These scales and items were designed based on multi-trait scaling analyses, reliability and validity tests, and patients' opinions through a semi-structured interview [1]. This questionnaire is also designed to evaluate symptoms associated with gastric involvement, a complication of corrosive ingestion [5,8]. We aimed to assess the performance of this questionnaire in Indian patients with corrosive-induced refractory esophageal strictures.

## Materials and methods

This was a single-center cross-sectional study. Data collection spanned six months in 2022. Consecutive patients with refractory or recurrent esophageal strictures due to corrosive ingestion who came to our center for outpatient esophageal dilation were recruited via opportunity sampling. Patients had to be above 18 years of age at the time of inclusion and literate in English or Hindi to be eligible. The participation of all patients was preceded by written informed consent. Patients were excluded if they had undergone reconstructive surgery of the upper GI tract, had a feeding gastrostomy/jejunostomy in place, or had been undergoing stricture dilation for less than 6 months.

We utilized the definition given by Kochman et al. for a refractory esophageal stricture: 'an anatomic restriction because of cicatricial luminal compromise or fibrosis that results in the clinical symptom of dysphagia in the absence of endoscopic evidence of inflammation, and a failure to dilate to a diameter of 14 mm over 5 endoscopic sessions at 2-week intervals' [18]. They defined recurrent esophageal stricture as 'an anatomic restriction because of cicatricial luminal compromise or fibrosis that results in the clinical symptom of dysphagia in the absence of endoscopic evidence of inflammation, and a failure to maintain a satisfactory luminal diameter for 4 weeks once the target diameter of 14 mm has been achieved' [18]. Stricture dilation was performed using Savary-Gilliard® dilators (Cook Medical®). Permission to use the EORTC QLQ-OG 25 questionnaire in English and its Hindi translation developed by the EORTC was obtained from the European Organisation for Research and Treatment of Cancer. All translation projects are coordinated by the EORTC translation team and follow a translation procedure. The translation procedure includes two back-translations to English and pilot testing [19].

Statistical analysis

Items were recorded on a 4-point Likert scale ranging from 1 to 4 (1= Not at all; 2= A little; 3= Quite a bit, 4= Very much) to indicate the frequency of symptoms in the past week. Higher scores indicated more symptoms and, thus, poorer quality of life. Raw scores for multi-item scales were calculated by averaging scores of all component items. Raw scores were transformed to a standard score in the 0-100 range as per the EORTC scoring manual for this questionnaire [20]. All analyses were performed using IBM SPSS Statistics for Windows, Version 25.0 (IBM Corp. Armonk, NY). A two-tailed p-value of <0.05 was considered significant.

EORTC QLQ-OG 25 HRQL scores

For each item on the questionnaire, mean, standard deviation (SD), and median were calculated for the standard scores. In addition, the raw scores were analyzed for the percentage frequency for each value (1-4), along with skewness and kurtosis. Item performance was assessed using floor and ceiling effects. Floor and ceiling effects are said to have occurred if a considerable proportion of respondents chose either the lowest or highest scores for a particular item. These indicate that extreme items must be added to the scale's lower or upper end, limiting content validity [21]. Thus, subjects at the lowest or highest score limits cannot be differentiated from each other, reducing reliability [21]. Additionally, floor and ceiling effects diminish the ability of the questionnaire to detect changes over time [21]. Typically, floor or ceiling effects above 15 % are considered significant [22].

Convergent and discriminant validity

A multi-trait scaling analysis was conducted to check if the existing multi-item scales were appropriately structured. This technique is based on item correlations and is frequently used to assess the structure of health-related quality-of-life measures [23]. The original six scales from the questionnaire were analyzed. A corrected item-total correlation (the correlation between an item and its scale when corrected for overlap) was used to determine convergent validity. A correlation of greater than 0.40 is recommended for each item [23]. According to Fayers and Manchin, scaling is considered successful if the corrected item-total correlations are significantly higher for an item and its scale compared to its correlation with other scales (divergent validity) [23]. Items that repeatedly correlate higher with other scales and weakly with their scale are not considered suitable for inclusion in their scale. 

Reliability

Reliability refers to the degree to which the results obtained by measurement and procedure can be replicated [24,25]. Reliability was measured as internal consistency using Cronbach's alpha. A value of >0.7 is recommended for comparing between groups [23,26].

## Results

A total of 31 participants were included in the study. Table 1 summarizes the descriptive characteristics.

**Table 1:** Descriptive characteristics of patients included in the study (n=31).

Parameter		
Age (in years)	Mean (SD)	30.7 (12.0)
Sex	Males	10 (32.3%)
	Females	21 (67.7%)
Language	English	5 (16.1%)
	Hindi	26 (83.9%)

The average time taken to complete the questionnaire was 6.70 minutes. There was no missing data. Table 2 displays the standard scores' mean, standard deviation, and median across the six and ten singe-item scales. The highest scores were seen with Eating in front of others (mean 58.06), anxiety (mean 51.61), Eating (mean 45.43), Weight loss (mean 44.09), and Dysphagia (mean 41.22). The trouble with taste (mean 4.30), trouble with coughing (mean 4.30), trouble with swallowing saliva (mean 9.68), and Trouble with talking (mean 11.83) scored the least.

**Table 2:** Mean, standard deviation, and median of standard scores across the six multi-item scales and ten single items of the EORTC QLQ-OG 25 (n=31). EORTC QLQ-OG 25: European organization Research and Treatment of Cancer Quality of Life Questionnaire-Oesophagogastric 25

Scale	Mean	SD	Median
Dysphagia	41.22	27.70	33.33
Eating	45.43	28.93	41.67
Reflux	18.28	27.67	0.00
Odynophagia	24.73	22.31	16.67
Pain and Discomfort	12.37	18.24	0.00
Anxiety	51.61	33.71	50.00
Eating with others	58.06	44.69	66.67
Dry mouth	13.98	26.91	0.00
Trouble with taste	4.30	14.25	0.00
Body image	32.26	37.99	0.00
Trouble with swallowing saliva	9.68	26.10	0.00
Choked when swallowing	36.56	38.83	33.33
Trouble with coughing	4.30	16.65	0.00
Trouble talking	11.83	27.95	0.00
Weight loss	44.09	38.86	33.33
Hair loss	19.35	31.94	0.00

Item performance

Table 3 shows the percentage frequencies of individual values for each item, skewness, and kurtosis. Floor effects were most prominent in items 18,15,17,19 (>80%); items 11, 14, 9, and 12 (>70%). Ceiling effects were highest with items 6, 22 (48.4%) and items 1, 4 (32.3%). The most skewed responses were seen in items 15, 18, 19, 11, and 14.

**Table 3:** Frequency of each value, skewness, and kurtosis of the responses to the 25 items of the EORTC QLQ-OG 25 (n=31). EORTC QLQ-OG 25: European organization Research and Treatment of Cancer Quality of Life Questionnaire-Oesophagogastric 25

Item no.	Question	Frequency of responses as percentages				Skewness	Kurtosis
		1	2	3	4		
1	Have you had problems eating solid foods?	9.7	25.8	32.3	32.3	-0.4	-0.9
2	Have you had problems eating liquidized or soft foods?	38.7	16.1	29.0	16.1	0.2	-1.4
3	Have you had problems drinking liquids?	64.5	19.4	6.5	9.7	1.5	1.3
4	Have you had trouble enjoying your meals?	32.3	29.0	6.5	32.3	0.3	-1.6
5	Have you felt full up too quickly after beginning to eat?	58.1	16.1	9.7	16.1	1.0	-0.5
6	Has it taken you a long time to complete your meals?	16.1	25.8	9.7	48.4	-0.4	-1.5
7	Have you had difficulty eating?	16.1	51.6	16.1	16.1	0.6	-0.5
8	Have you had acid indigestion or heartburn?	64.5	16.1	9.7	9.7	1.4	0.7
9	Has acid or bile coming into your mouth be a problem?	71.0	16.1	9.7	3.2	1.8	2.4
10	Have you had discomfort when eating?	22.6	51.6	16.1	9.7	0.7	0.1
11	Have you had pain when you eat?	77.4	12.9	6.5	3.2	2.3	4.8
12	Have you had pain in your stomach area?	71.0	22.6	6.5	0.0	1.6	1.5
13	Have you had discomfort in your stomach area?	67.7	25.8	6.5	0.0	1.4	1.0
14	Have you had a dry mouth?	74.2	12.9	9.7	3.2	1.9	2.8
15	Have you had problems with your sense of taste?	90.3	6.5	3.2	0.0	3.6	13.0
16	Have you choked when swallowing?	38.7	35.5	3.2	22.6	0.7	-0.9
17	Have you had difficulty swallowing your saliva?	83.9	9.7	0.0	6.5	3.0	8.5
18	Have you coughed?	93.5	0.0	6.5	0.0	3.7	12.7
19	Have you had difficulty talking?	80.6	9.7	3.2	6.5	2.5	5.4
20	Have you been thinking about your illness?	16.1	45.2	16.1	22.6	0.3	-1.0
21	Have you worried about your health in the future?	22.6	19.4	29.0	29.0	-0.2	-1.3
22	Have you had trouble with eating in front of other people?	29.0	16.1	6.5	48.4	-0.3	-1.8
23	Have you worried about your weight being too low?	29.0	35.5	9.7	25.8	0.4	-1.3
24	Have you felt physically less attractive due to your disease or treatment?	51.6	12.9	22.6	12.9	0.6	-1.1
25	Answer this question only if you lost any hair: If so, were you upset by the loss of your hair?	67.7	12.9	12.9	6.5	1.5	0.9

Convergent and discriminant validity

The six multi-item scales are analyzed in Table 4, along with Item-other scale correlations for all 25 items. Corrected item-total correlations were all above the minimum acceptable of 0.4 except for odynophagia (0.328) and item 3 of dysphagia (0.344). Item-other domain correlations revealed significantly high correlations between the Anxiety domain and the first two items of Dysphagia: Pearson's coefficients(r) of 0.60 and 0.56, respectively (p-values <0.001). Item-other domain correlations for both items of the Odynophagia scale were high: item 10 was highly correlated with the Eating scale (r-value = 0.71, p-value <0.001), and item 11 was highly correlated with the Pain and Discomfort Scale (r=0.60, p-value <0.001).

Reliability

Table 4 displays Cronbach's alpha for the six multi-item scales. Cronbach's alpha was >0.7 for all scales except odynophagia (0.49).

**Table 4:** Cronbach’s alpha and the Item-total correlations for the six multi-item scales and item-other scale correlations for all 25 items (n=31).

Scale	Questions	Corrected Item-total correlations	Cronbach's alpha for the scale	Item-other scale correlation
Dysphagia	Have you had problems eating solid foods?	0.55	0.71	-0.03 to 0.61
	Have you had problems eating liquidized or soft foods?	0.74		-0.05 to 0.56
	Have you had problems drinking liquids?	0.34		0.04 to 0.58
Eating	Have you had trouble enjoying your meals?	0.44	0.75	-0.11 to 0.39
	Have you felt full up too quickly after beginning to eat?	0.54		0.06 to 0.42
	Has it taken you a long time to complete your meals?	0.66		0.21 to 0.67
	Have you had difficulty eating?	0.6		0.21 to 0.67
Reflux	Have you had acid indigestion or heartburn?	0.65	0.77	0.03 to 0.64
	Has acid or bile coming into your mouth been a problem?	0.65		-0.19 to 0.40
Odynophagia	Have you had discomfort when eating?	0.33	0.49	0.13 to 0.71
	Have you had pain when you eat?	0.33		-0.12 to 0.60
Pain and Discomfort	Have you had pain in your stomach area?	0.6	0.75	-0.15 to 0.68
	Have you had discomfort in your stomach area?	0.6		-0.04 to 0.45
Anxiety	Have you been thinking about your illness?	0.74	0.85	-0.01 to 0.67
	Have you worried about your health in the future?	0.74		-0.02 to 0.55
Dry mouth	Have you had a dry mouth?			-0.15 to 0.31
Trouble with taste	Have you had problems with your sense of taste?			-0.08 to 0.48
Trouble swallowing saliva	Have you had difficulty swallowing your saliva?			-0.10 to 0.55
Choked when following	Have you choked when swallowing?			-0.01 to 0.55
Trouble talking	Have you had difficulty talking?			0.02 to 0.68
Trouble with coughing	Have you coughed?			-0.16 to 0.68
Eating with others	Have you had trouble with eating in front of other people?			0.11 to 0.65
Weight loss	Have you worried about your weight being too low?			-0.15 to 0.68
Body image	Have you felt physically less attractive as a result of your disease or treatment?			-0.13 to 0.68
Hair loss	Answer this question only if you lost any hair: If so, were you upset by the loss of your hair?			-0.16 to 0.69

## Discussion

Health-related QoL is an essential tool for collecting patient-reported outcomes for chronic diseases. Objectively measured clinical data are helpful for clinicians but may not match patients' functional capacity and well-being: outcomes of greater value from a patient's point of view [27]. Patients with refractory corrosive-induced esophageal strictures can suffer from poor quality of life due to multiple factors and, along with clinicians, are faced with the difficult choice of continuing with periodic dilation or pursuing complex reconstructive GI surgery. Here, we studied the performance of an existing cancer QoL questionnaire as a potential HRQoL tool in this patient population.

Scores revealed that trouble with taste, coughing, swallowing saliva, and talking were the least bothersome symptoms for patients. High floor effects in these four items (>80%) and the highly skewed response distribution (skewness >+2 and kurtosis >+3) indicate that these are not usual complaints in this population and are not being reliably measured. Pain is also not a common complaint given the high floor effects in pain during eating and pain in the stomach area. This is similar to scores in patients with esophagogastric cancer [1]. Ceiling effects were not as prominent as floor effects nor skewed, with the highest effect seen with item 6 (long time completing meals) and item 22 (eating with others).

Odynophagia's low corrected item-total correlation (0.33) suggests that combining the two questions on discomfort and pain during eating into a single scale may not be helpful. They correlate strongly with two other separate scales. The corrected item-total correlation for the item on difficulty swallowing liquids on the Dysphagia scale is also low (0.34). This may be explained by the mechanical nature of the dysphagia of corrosive esophageal strictures, as most respondents did not have trouble swallowing liquids.

Five of the six scales were reliable, except the Odynophagia scale (alpha of 0.49). This can be explained by the differences in responses to its component questions. Most subjects denied pain while eating by responding with 1 (77.4%), in contrast to the majority who chose 2 or above (77.4%) in response to discomfort.

A study by Anand et al. evaluated the QoL of a similar patient population of corrosive-induced esophageal strictures being managed by endoscopic dilation at a tertiary care center in North India [17]. They used the World Health Organization Quality of Life Brief Version (WHOQoL-BREF) tool for QoL and measured dysphagia, psychological morbidity, and Disability (WHODAS 2.0) separately. The latter three, including dysphagia, were found to have strong correlations with quality of life. This indicates that QoL is an excellent multi-dimensional measure of patient outcomes in patients with esophageal strictures on long-term endoscopic dilation. The advantage that EORTC QLQ-OG 25 has is an integrated Dysphagia scale, thus eliminating the need for a separate measure of dysphagia. WHOQoL-BREF's advantage is its questions related to social relationships and the environment.

Raynaud et al. utilized the EORTC QLQ-OG 25 in France to compare the longitudinal change in the quality of life of patients with acute severe caustic ingestion managed conservatively with those managed surgically [4]. They captured a statistically significant change in the Dysphagia scale with a sample size of 21, an encouraging functional application of this questionnaire demonstrating its ability to capture longitudinal change in this population.

Faron et al. evaluated the QoL of 134 patients in France with a history of caustic ingestion using the EORTC QLQ-OG 25, the Short Form 12 (SF-12), and the Hospital Anxiety and Depression Scale (HAD) questionnaires [3]. 74% of the patients had undergone esophageal reconstruction. They found significant correlations between the EORTC QLQ-OG 25 and the general questionnaires SF-12 and HAD. However, the correlation was lower than the correlations between SF-12 and HAD. Three patients with complete functional failure and a lifelong inability to eat had normal SF-12 and HAD scores but low EORTC QLQ-OG 25 scores. Based on these observations, the investigators concluded that QoL, as measured by QLQ-OG 25, heavily emphasizes functionality (ability to eat) that may not affect general aspects of QoL.

However, as in our study, this may not be the case for patients who rely on oral intake for nutrition and have refractory strictures requiring periodic dilation. Their study reveals differences in QLQ-OG 25 scores between those defined as treatment success and those as treatment failure. The latter scored significantly higher on the Eating with Others (psychosocial component) scale. Additionally, our study showed a significantly high correlation (>0.5, p<0.001) between anxiety and the first item of dysphagia. Thus indicating that functionality can affect psychosocial aspects in the studied population.

A small sample size limits this study. The performance of the single items cannot be satisfactorily commented on in the absence of test-retest reliability, which also limits the assessment of the ability of the questionnaire to measure change over time. A more extensive study incorporating test-retest reliability is desirable for the future. The time frame for data collection was relatively short, at six months. It raises the potential for selection bias toward recruiting patients with shorter dilation intervals and, thus, greater disease severity. Lastly, in patients who have had an objectively successful treatment or those who do not rely on oral intake for nutrition, symptoms of eating disorders may play a less critical role. General QoL tools, unlike the QLQ-OG 25, could provide a better assessment.

## Conclusions

The EORTC QLQ-OG 25 has good internal consistency, convergent validity, and discriminant validity in patients with refractory/ recurrent corrosive-induced benign esophageal strictures. It can be satisfactorily used for evaluating QoL in these patients. However, the Odynophagia scale had low internal consistency, convergent validity, and discriminant validity due to a combination of questions that correlate poorly with each other and correlate better with other scales. Prominent floor effects and highly skewed responses to questions evaluating taste, cough, swallowing saliva, and talking indicate that these items are not particularly relevant to this population.

## References

[1] Lagergren P, Fayers P, Conroy T (2007). Clinical and psychometric validation of a questionnaire module, the EORTC QLQ-OG25, to assess health-related quality of life in patients with cancer of the oesophagus, the oesophago-gastric junction and the stomach. Eur J Cancer.

[2] Aaronson NK, Ahmedzai S, Bergman B (1993). The European Organization for Research and Treatment of Cancer QLQ-C30: a quality-of-life instrument for use in international clinical trials in oncology. J Natl Cancer Inst.

[3] Faron M, Corte H, Poghosyan T (2021). Quality of life after caustic ingestion. Ann Surg.

[4] Raynaud K, Seguy D, Rogosnitzky M, Saulnier F, Pruvot FR, Zerbib P (2016). Conservative management of severe caustic injuries during acute phase leads to superior long-term nutritional and quality of life (QoL) outcome. Langenbecks Arch Surg.

[5] Contini S, Scarpignato C (2013). Caustic injury of the upper gastrointestinal tract: a comprehensive review. World J Gastroenterol.

[6] Lakshmi CP, Vijayahari R, Kate V, Ananthakrishnan N (2013). A hospital-based epidemiological study of corrosive alimentary injuries with particular reference to the Indian experience. Natl Med J India.

[7] Kalayarasan R, Ananthakrishnan N, Kate V (2019). Corrosive ingestion. Indian J Crit Care Med.

[8] Chirica M, Bonavina L, Kelly MD, Sarfati E, Cattan P (2017). Caustic ingestion. Lancet.

[9] van Boeckel PG, Siersema PD (2015). Refractory esophageal strictures: what to do when dilation fails. Curr Treat Options Gastroenterol.

[10] Everett SM (2019). Endoscopic management of refractory benign oesophageal strictures. Ther Adv Gastrointest Endosc.

[11] Broor SL, Kumar A, Chari ST (1989). Corrosive oesophageal strictures following acid ingestion: clinical profile and results of endoscopic dilatation. J Gastroenterol Hepatol.

[12] Broor SL, Raju GS, Bose PP, Lahoti D, Ramesh GN, Kumar A, Sood GK (1993). Long term results of endoscopic dilatation for corrosive oesophageal strictures. Gut.

[13] Arunachalam R, Rammohan A (2016). Corrosive injury of the upper gastrointestinal tract: a review. Arch Clin Gastroenterol.

[14] Contini S, Swarray-Deen A, Scarpignato C (2009). Oesophageal corrosive injuries in children: a forgotten social and health challenge in developing countries. Bull World Health Organ.

[15] Bonavina L, Chirica M, Skrobic O (2015). Foregut caustic injuries: results of the world society of emergency surgery consensus conference. World J Emerg Surg.

[16] Chen YJ, Seak CJ, Chen CC (2020). The association between caustic ingestion and psychiatric comorbidity based on 396 adults within 20 years. Risk Manag Healthc Policy.

[17] Anand N, Sharma A, Shah J, Kochhar R, Singh SM (2021). Quality of life in patients of corrosive esophageal stricture treated with endoscopic dilatation. JGH Open.

[18] Kochman ML, McClave SA, Boyce HW (2005). The refractory and the recurrent esophageal stricture: a definition. Gastrointest Endosc.

[19] Kulis D, Bottomley A, Velikova G, Greimel E, Koller M (2017). EORTC Quality of Life Group Translation Procedure, Fourth Edition. https://www.eortc.org/app/uploads/sites/2/2018/02/translation_manual_2017.pdf.

[20] Fayers PM, Aaronson NK, Bjordal K, Groenvold M, Curran D, Bottomley A (2001). The EORTC QLQ-C30 Scoring Manual (3rd Edition). European Organisation.

[21] Terwee CB, Bot SD, de Boer MR (2007). Quality criteria were proposed for measurement properties of health status questionnaires. J Clin Epidemiol.

[22] McHorney CA, Tarlov AR (1995). Individual-patient monitoring in clinical practice: are available health status surveys adequate?. Qual Life Res.

[23] Fayers PM, Machin D (2007). Quality of Life: The Assessment, Analysis and Interpretation of Patient-Reported Outcomes. Second.

[24] Bolarinwa OA (2015). Principles and methods of validity and reliability testing of questionnaires used in social and health science researches. Niger Postgrad Med J.

[25] Rothman Rothman, K K, Greenland Greenland, S S, Lash Lash, T T (2008). Modern Epidemiology, 3rd Edition. https://www.rti.org/publication/modern-epidemiology-3rd-edition.

[26] Reeve BB, Wyrwich KW, Wu AW (2013). ISOQOL recommends minimum standards for patient-reported outcome measures used in patient-centered outcomes and comparative effectiveness research. Qual Life Res.

[27] Guyatt GH, Feeny DH, Patrick DL (1993). Measuring health-related quality of life. Ann Intern Med.

